# The Flavonoid Jaceosidin from* Artemisia princeps* Induces Apoptotic Cell Death and Inhibits the Akt Pathway in Oral Cancer Cells

**DOI:** 10.1155/2018/5765047

**Published:** 2018-05-13

**Authors:** Hye-Yeon Han, Hyung Joon Kim, Seung-Hwa Jeong, Jiyeon Kim, Sung-Hee Jeong, Gyoo Cheon Kim, Dae-Seok Hwang, Uk-Kyu Kim, Mi Heon Ryu

**Affiliations:** ^1^Department of Oral Pathology, School of Dentistry, Research Institute for Oral Biotechnology, Pusan National University, Yangsan, Gyeongnam 50612, Republic of Korea; ^2^Department of Oral Physiology, BK21 Plus Project and Institute of Translational Dental Sciences, School of Dentistry, Pusan National University, Yangsan, Gyeongnam 50612, Republic of Korea; ^3^Department of Preventive and Community Dentistry, BK21 Plus Project, School of Dentistry, Pusan National University, Yangsan, Gyeongnam 50612, Republic of Korea; ^4^Department of Pediatric Dentistry, School of Dentistry, Dental Research Institute, Pusan National University, Yangsan, Gyeongnam 50612, Republic of Korea; ^5^Department of Oral Medicine, School of Dentistry, Pusan National University, Dental Research Institute, Yangsan, Gyeongnam 50612, Republic of Korea; ^6^Department of Dental Anatomy, BK21 Plus Project, School of Dentistry, Pusan National University, Yangsan, Gyeongnam 50612, Republic of Korea; ^7^Department of Oral and Maxillofacial Surgery, School of Dentistry, Pusan National University, Yangsan, Gyeongnam 50612, Republic of Korea; ^8^Department of Oral Pathology, BK21 Plus Project, School of Dentistry, Pusan National University, Yangsan, Gyeongnam 50612, Republic of Korea

## Abstract

Jaceosidin is a single compound from the Japanese mugwort* Artemisia princeps*, which is used as a food and a traditional medicinal herb.* A. princeps* extracts and flavonoid components have been shown to have antihyperglycaemic, antioxidant, and anti-inflammatory properties. Although the anticancer properties of these extracts were recently demonstrated, the related mechanisms have not been characterised. In this study, we investigated the effects of jaceosidin in oral squamous cell carcinoma (OSCC) cells and initially showed selective suppression of proliferation (IC_50_ = 82.1 *μ*M in HSC-3 cells and 97.5 *μ*M in Ca9.22 cells) and accumulation of cells at the sub-G1 stage of the cell cycle. In addition, jaceosidin increased cleavage of caspase-9 and caspase-3 in OSCC cells, although caspase-8 was not detected. In further experiments, jaceosidin downregulated Akt phosphorylation and ectopic activation of Akt blocked the antiproliferative effects of jaceosidin. Finally, we showed that jaceosidin has no effects on HaCaT normal epithelial cell viability, indicating selective chemotherapeutic potential of jaceosidin and that tumour-specific downregulation of Akt increases apoptosis and inhibits growth in OSCC cells.

## 1. Introduction

Oral squamous cell carcinoma (OSCC) accounts for 90% of oral cavity malignancies, and about 3,000 new OSCC diagnoses were recorded in 2011 in South Korea [1]. Surgical resection and chemotherapy are the prevailing therapeutic strategies for OSCC, and although these treatments have improved over several decades, survival rates of OSCC patients remain relatively low at around 50% [2]. In addition, defects of head and neck regions following removal of OSCCs are aesthetically and functionally debilitating and include mastication problems and difficulties speaking. Chemotherapies for OSCC patients also have severe side effects, such as nausea/vomiting, alopecia, mucositis, headaches, chronic weakness, and exhaustion, and these contribute to low treatment success rates [3]. Therefore, targeted chemotherapeutic agents for OSCC are required to reduce complications and improve 5-year survival rates. Such anticancer agents should primarily act by inducing apoptosis, because dysfunctional apoptotic pathways are the hallmark of all malignancies [4].

*Artemisia* genus consists of a large and diverse family of plants, and more than 500* Artemisia *herbs are widespread worldwide and many are endemic to Korea [4]. Most* Artemisia* species have been used as food additives and teas and provide important ingredients for various traditional Korean medicines for gynecologic diseases, gastrointestinal conditions, hepatitis, eczema, furuncles, inflammation, and tumours [4, 5]. Extracts from* Artemisia* species contain many flavonoids, including jaceosidin, eupalitin, and eupafolin [6], and jaceosidin is considered one of the most active ingredients [7–9].

Few studies demonstrate the antioxidant, anti-inflammatory, and immunosuppressive effects of jaceosidin and inhibition of several human cancers. However, jaceosidin promoted angiogenesis in endothelial cells [10] and had strong antimutagenic activity and anticancer chemotherapeutic potential [9] in previous studies. Furthermore, jaceosidin reportedly inhibited the functions of E6 and E7 oncoproteins of human papillomavirus 16, potentially limiting the proliferation of several human cancer cells [9, 11]. Accordingly, Chung et al. showed that ethanol extracts of* Artemisia* inactivate colitis-associated colon tumorigenesis in mice [12]. However, to our knowledge, the effects of jaceosidin on OSCCs have not been investigated to date. Thus, we demonstrated the pharmacological potential and modes of action of jaceosidin in OSCC cells.

## 2. Materials and Methods

### 2.1. Reagents and Antibodies

Jaceosidin (CAS 18085-97-7) was kindly provided by Professor Hyungwoo Kim of the Pusan National University. Paclitaxel, propidium iodide (PI), DAPI, and 3,4,5-dimethyl-N-methylthiazol-2-yl-2,5-d-phenyl tetrazolium bromide (MTT) were purchased from Sigma-Aldrich (St. Louis, MO, USA). Annexin V-FITC apoptosis detection kits were obtained from BD Biosciences (CA, USA) and antibodies against cleaved caspase-3, cleaved caspase-9, cleaved poly(ADP-ribose) polymerase (PARP), Akt, and phospho-Akt were supplied by Cell Signaling Technology (Beverly, MA, USA). Anti-mouse IgG secondary antibody and anti-rabbit IgG secondary antibody were obtained from Enzo Life Sciences (Farmingdale, NY, USA), whereas anti-beta-actin antibody was purchased from Santa Cruz Biotechnology (Santa Cruz, CA, USA).

### 2.2. Cell Culture

The human OSCC cell lines HSC-3 and Ca9.22 were obtained from the Japanese Collection of Research Bioresources Cell Bank (JCRB Cell Bank). HSC-3 and HaCaT cells were cultured in Dulbecco's Modified Eagle's Medium (DMEM) containing 10% fetal bovine serum and 1% penicillin/streptomycin in a humidified environment containing 5% CO_2_ at 37°C. Ca9.22 cells were cultured and maintained in Modified Eagle's Medium (MEM, Hyclone, UT, USA) containing 10% fetal bovine serum (FBS, Hyclone) with 1% penicillin/streptomycin (Invitrogen, NY, USA) in a humidified chamber containing 5% CO_2_/95% air at 37°C. Equal numbers of OSCC cells (4 × 10^4^ cells/well) were seeded onto 24-well plates and were used in MTT assays and other analyses as adherent cultures.

### 2.3. Proliferation Assays

The antiproliferative activities of jaceosidin were determined using MTT assays. Briefly, cells were seeded at 4 × 10^4^ cells per well in 24-well plates and were allowed to adhere overnight. Jaceosidin was diluted in DMSO and was added to cell cultures at 0, 12.5, 25, 50, or 100 *μ*M. After 24 h jaceosidin treatments, MTT solution (500 *μ*l) was added to cells and incubated at 37°C for 4 h in air containing 5% CO_2_. Media were then carefully removed after solubilisation of formazan crystals, and optical densities (OD) of the converted dye were measured at 570 nm using a microplate reader (Bio-Rad Laboratories, Hercules, CA, USA).

### 2.4. Cell Morphology

To assess morphological changes of HSC-3 and Ca9.22 cells after jaceosidin treatments, cells were analysed and photographed using a phase contrast microscope at 200x magnification (Olympus, Tokyo, Japan).

### 2.5. Cell Cycle Analyses

Changes in cell cycle progression of jaceosidin-treated OSCC cells were assessed using flow cytometry. In these analyses, HSC-3 and Ca9.22 cells (1 × 10^6^ cells per well) were treated with DMSO (vehicle) or jaceosidin at 25, 50, or 100 *μ*M for 48 h. Cells were then harvested and stained with PI solution (10 *μ*g/ml). Cell cycle stages were then evaluated using a FACS Scan flow cytometer (BD Biosciences, Heidelberg, Germany).

### 2.6. Assessments of Apoptosis

To determine rates of jaceosidin-induced apoptosis, cells were labelled with Annexin V-FITC and PI and were then analysed using flow cytometry. Cells were seeded in 6-well plates at 3 × 10^5^ cells per well and were incubated overnight and then treated with jaceosidin at 25, 50, or 100 *μ*M for 48 h. Control cells were treated with vehicle (DMSO) for 48 h and cells treated with 30 nM paclitaxel for 48 h were used as positive controls. After treatments, cells were harvested with trypsin, washed with phosphate-buffered saline (PBS) twice, resuspended in 500 *μ*L of Binding Buffer (Annexin V-FITC apoptosis detection kit, Enzo), and finally stained using Annexin V-FITC apoptosis detection kits (Enzo) at room temperature for 5 min in the dark according to the manufacturer's instructions. Stained cells were then analysed using a FACS Scan flow cytometer (BD Biosciences, Heidelberg, Germany), and the data were processed using FACSCanto II software.

### 2.7. Western Blotting

Following jaceosidin treatments, cell lysates were extracted using RIPA buffer (Cell Signaling Technology) according to the manufacturer's instructions and protein concentrations were determined. Subsequently, 40 *μ*g samples were electrophoresed on SDS-polyacrylamide gels in triplicate and were then transferred to polyvinylidene fluoride membranes. After blocking with skim milk, membranes were incubated with primary antibodies against cleaved caspase-3, cleaved caspase-9, cleaved PARP, Akt, phospho-Akt, and beta-actin (internal control) at 4°C overnight. Finally, HRP-conjugated secondary antibodies (1 : 5000) were applied at room temperature and protein signals were detected using SuperSignal West-Femto reagent (Pierce, Rockford, IL, USA).

### 2.8. Statistical Analysis

Data are presented as means ± standard deviations (SD). Differences between control and treatment groups were identified using Student's *t*-test (SPSS ver. 21.0, SPSS, Illinois, USA) and were considered significant when *p* < 0.05, as indicated with asterisks.

## 3. Results

### 3.1. Jaceosidin Inhibits OSCC Cell Proliferation

**Figure 1:**
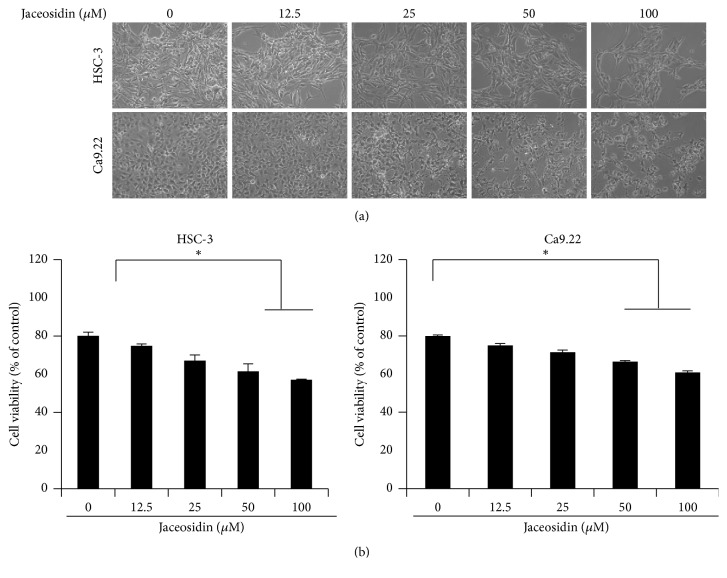
Antiproliferative effects of jaceosidin in OSCC cells; HSC-3 and Ca9.22 OSCC cells were treated with jaceosidin at 0, 12.5, 25, 50, or 100 *μ*M for 24 h. (a) Morphologies of jaceosidin-treated HSC-3 cells (top) and Ca9.22 cells (bottom). (b) Percentages of viable cells were determined after 24 h using MTT assays. The viability of HSC-3 cells (left) and Ca9.22 cells (right); independent experiments were performed in triplicate; ^*∗*^*p* < 0.05.

The anticancer effects of jaceosidin against the OSCC cells lines HSC-3 and Ca9.22 were evaluated using MTT assays. Jaceosidin inhibited OSCC cell proliferation in a dose-dependent manner (Figures 1(a) and 1(b)), with half inhibitory concentrations (IC_50_) of 82.1 *μ*g/mL in HSC-3 cells and 97.5 *μ*g/mL in Ca9.22 cells. In addition, marked morphological changes were evident following treatments of OSCC cells with jaceosidin. Specifically, jaceosidin-treated OSCC cells were smaller and rounder and lost visible cellular processes in a dose-dependent manner (Figure 1(a)), suggesting that jaceosidin has antiproliferative activity in oral cancer cells.

### 3.2. Jaceosidin Induced Early and Late Apoptosis in OSCC Cells and Caused Accumulation of Cells in the Sub-G1 Phase

**Figure 2:**
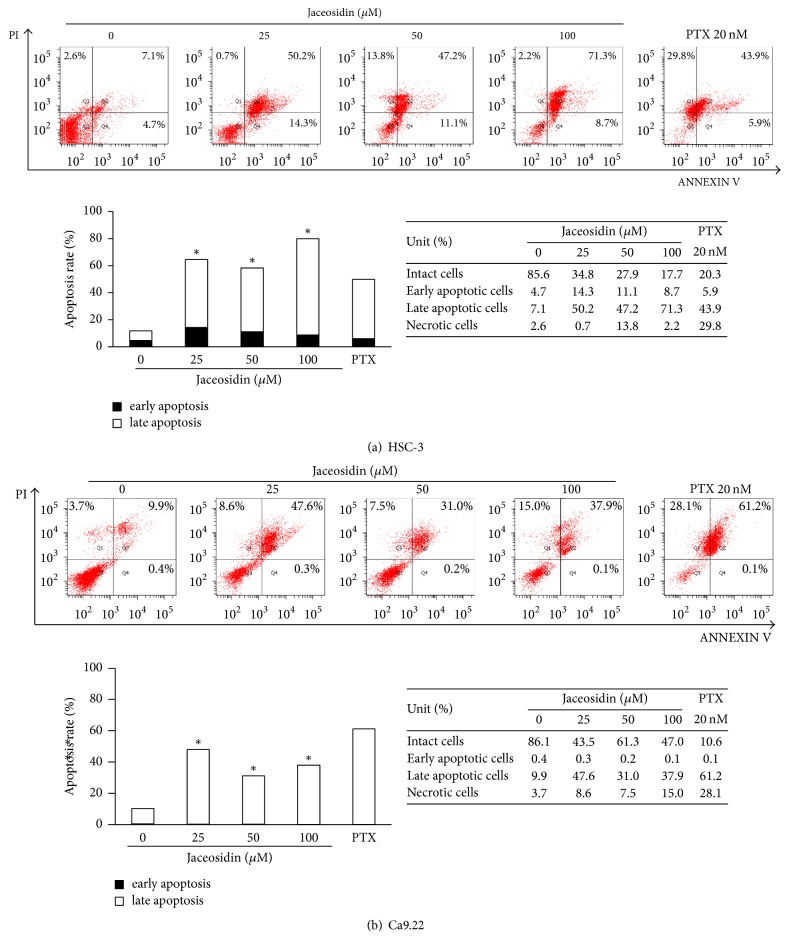
Jaceosidin induces early and late apoptosis in OSCC cells. Thus, to evaluate jaceosidin-mediated apoptosis, jaceosidin-treated OSCC cells (for 48 h) were labelled with PI and Annexin V and apoptotic staining profiles were analysed using flow cytometry (^*∗*^*p* < 0.05). Paclitaxel (PTX) was used as a positive control; (a) HSC-3 cells; (b) Ca9.22 cells.

**Figure 3:**
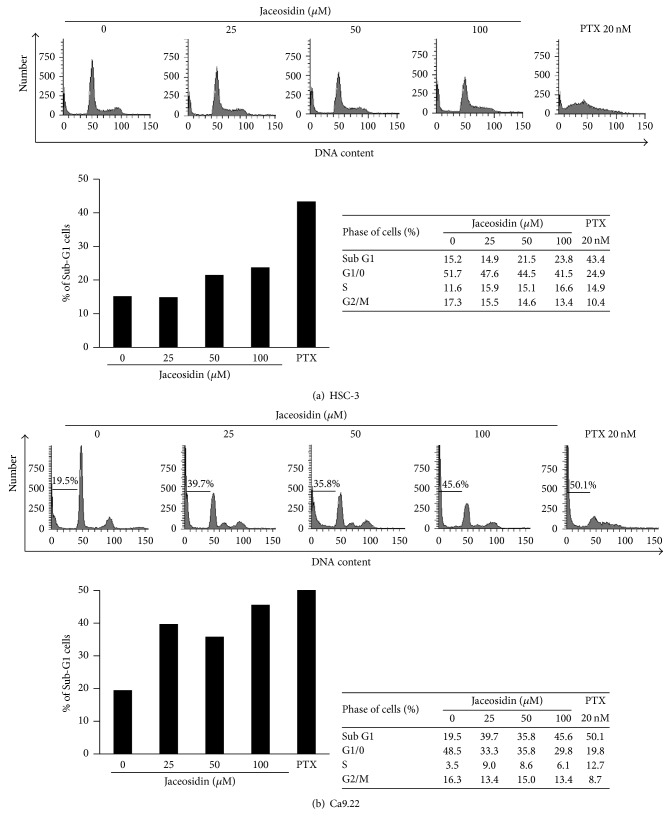
Jaceosidin-treated OSCC cells showed cell cycle arrest at the sub-G1 phase; OSCC cells were cultured in the presence of the indicated jaceosidin concentrations for 48 h and were stained with propidium iodide (PI). Cell cycle distributions were then evaluated using flow cytometry with paclitaxel (PTX) treatments as a positive control for cell cycle arrest; (a) HSC-3 cells; (b) Ca9.22 cells.

We performed PI and Annexin V-FITC double staining to investigate the antiproliferative mechanisms of jaceosidin. Jaceosidin treatments for 48 h significantly increased the number of apoptotic cells compared with those of untreated cells, and these effects were dose-dependent. In particular, 25 *μ*M jaceosidin led to prominent early and late apoptosis in HSC-3 and Ca9.22 cells (Figures 2(a) and 2(b)), and marked increases in PI/Annexin V double positive cell numbers were observed relative to PI-negative/Annexin V-positive cell numbers. In addition, 100 *μ*M jaceosidin treatments led to marked increases in percentages of cells in the sub-G1 cell cycle stage (Figures 3(a) and 3(b)), with 23.8% and 45.6% sub-G1 accumulations of HSC-3 and Ca9.22 cells, respectively.

### 3.3. Jaceosidin Induces Cleavage of Caspase-9, PARP, and Caspase-3 and Inhibits Phosphorylation of Akt

**Figure 4:**
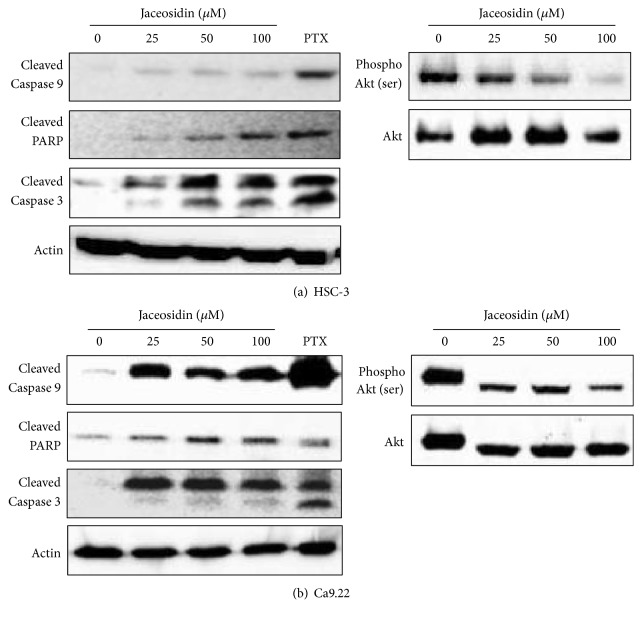
Jaceosidin triggers cleavage of caspase-9, PARP, and caspase-3 and decreases Akt phosphorylation; OSCC cells were treated with the indicated concentrations of jaceosidin for 48 h and cleaved caspase-9, PARP, and caspase-3 proteins and phosphorylated Akt were examined using western blotting. Paclitaxel (PTX) was used as a positive control for caspase and PARP cleavage; (a) HSC-3 cells; (b) Ca9.22 cells.

To characterise jaceosidin-mediated apoptotic signalling pathways, we determined the effects on caspase-9, PARP, and caspase-3 cleavage and phosphorylation. In the presence of jaceosidin, caspase-9, PARP, and caspase-3 cleavage was significantly increased in a dose-dependent manner (Figures 4(a) and 4(b), left). Cleaved caspase-3 is considered a biomarker of apoptosis, and Akt, also known as protein kinase B (PKB), has well characterised essential roles in tumour cell proliferation and survival [13, 14]. Accordingly, phosphorylation levels of Akt were decreased in jaceosidin-treated OSCC cells (Figure 4(a), right) compared with those in control OSCC cells (Figure 4(b), right), which had sustained high levels of phosphorylated Akt. These data indicate that jaceosidin-induced cell death is mediated by caspase pathway activation and blockade of Akt phosphorylation in OSCC cells.

### 3.4. Jaceosidin Does Not Affect Normal Epithelial Cell Proliferation

**Figure 5:**
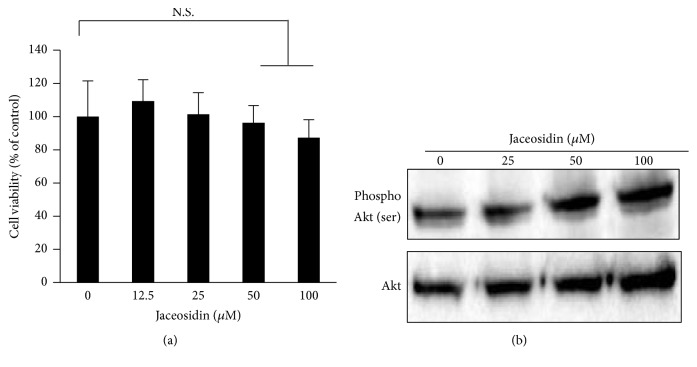
Cytotoxic effects of jaceosidin are absent in normal epithelial keratinocyte cells; (a) HaCaT cells were cultured in the presence of the indicated doses of jaceosidin for 24 h and cell viability was determined using MTT assays (NS: not significant). (b) HaCaT cells were cultured as in Figure 4 and phosphor-Akt levels were determined using western blotting.

To determine the effects of jaceosidin on normal cells, we performed proliferation assays in jaceosidin-treated HaCaT normal epithelial keratinocytes. As shown in Figure 5(a), jaceosidin did not inhibit HaCaT cell proliferation at doses of 12.5–100 *μ*M and had no effects on Akt phosphorylation (Figure 5(b)), indicating that the cytotoxic effects of jaceosidin are OSCC cancer cell-selective.

### 3.5. Akt Activation Reversed the Antiproliferative Effects of Jaceosidin in OSCC Cells

**Figure 6:**
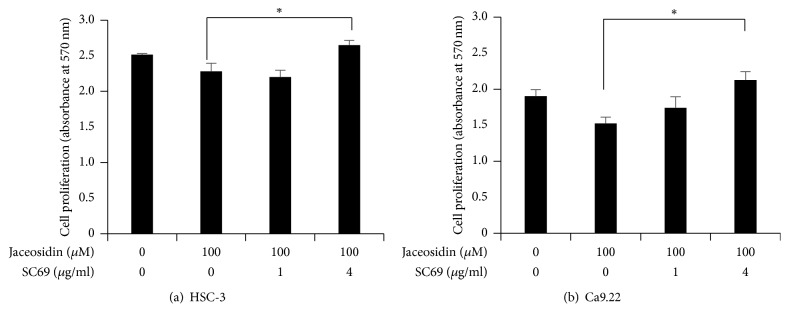
Activation of the Akt pathway reverses the antiproliferative effects of jaceosidin in OSCC cells; OSCC cells were treated with 100 *μ*M jaceosidin with the indicated concentrations of the Akt activator SC69 for 24 h and cell proliferation was determined using MTT assays; ^*∗*^*p* < 0.05; (a) HSC-3 cells; (b) Ca9.22 cells.

To determine the contributions of Akt pathway inactivation to the antiproliferative effects of jaceosidin, we cultured OSCC cells in the presence of 100 *μ*M jaceosidin and determined whether the Akt activator SC69 can restore proliferation. As shown in Figure 1, jaceosidin significantly inhibited OSCC cell proliferation at 100 *μ*M. However, OSCC cell proliferation was dose-dependently restored by SC69 treatments (Figure 6), suggesting that inhibition of the Akt pathway is central to the antiproliferative effects of jaceosidin.

## 4. Discussion

Various natural products have long been considered as sources of anticancer agents, and phytochemicals and other compounds from plants with food or traditional medicine uses have emerged as promising anticancer treatments and adjuvants. These components can be used to complement or moderate the side effects of anticancer drugs [15]. Moreover, the widely used anticancer agents paclitaxel, curcumin, and evodiamine were originally extracts from herbs and plants [15].

The present data demonstrate that jaceosidin treatments inhibit the proliferation of OSCC cells and cause morphological changes, with no effects on the proliferation of HaCaT cells at up to 100 *μ*M, suggesting tumour-specific cytotoxicity. Moreover, the ensuing mechanisms were indicated by increased accumulation of cells in the sub-G1 cell cycle stage, increased presence of early and late apoptotic cells, and cleavage of caspase-9, caspase-3, and PARP proteins. Taken together, these observations show that jaceosidin induces apoptosis of OSCC cells by activating intrinsic apoptotic pathways and also inactivates the Akt pathway.

Previous studies show that jaceosidin affects the proliferation of ovarian, bladder, and endometrial cancer cells [16–18], and most of these reports suggest that anticancer mechanisms of* Artemisia* extracts involve the induction of apoptosis. Moreover, in agreement with the present observations, Khan et al. showed that jaceosidin-induced apoptosis is accompanied by cell cycle arrest at the G2/M phase in U87 glioblastoma cells [19]. These data suggest that jaceosidin induces apoptosis by disrupting the cell cycle. Jaceosidin also reportedly induces apoptosis by altering mitochondrial membrane potential, and Lv et al. showed that jaceosidin induces apoptosis in human ovary cancer cells through mitochondrial pathways [18]. Only one previous report indicates extrinsic and intrinsic pathway activation during jaceosidin-induced apoptosis, suggesting that intrinsic pathways of apoptosis and cell cycle arrest are the primary anticancer mechanisms of jaceosidin.

Importantly, our results demonstrate that jaceosidin-induced cell death is confined to HSC-3 and Ca9.22 cancer cells, with no growth inhibition of HaCaT normal keratinocytes at concentrations that were antiproliferative in OSCC cells. In particular, no cytotoxic effects were observed in HaCaT cells at IC_50_ of jaceosidin in OSCC cells. The present data are also the first to show that jaceosidin-induced OSCC cell death is apoptotic, and no apoptotic indications were observed in HaCaT cells in the presence of the same concentrations of jaceosidin.

Apoptosis is regulated by complex caspase cascades, which are extrinsic and intrinsic. Caspase-3 plays key roles in the execution of apoptotic pathways, and as the end point of both intrinsic and extrinsic pathways, cleaved caspase-3 activates PARP and induces apoptosis [20, 21]. Our experiments show that jaceosidin treatment activates caspase-3 and cleavage of caspase-9, which is activated in the intrinsic apoptosis pathway. Jaceosidin also provoked the cleavage of caspases in HSC3 and Ca9.22 cells, suggesting that jaceosidin-induced apoptosis is caspase-dependent.

Although jaceosidin induces apoptosis via the intrinsic pathway, the precise mechanisms remain unclear. The Akt pathway has been associated with cell survival and proliferation in multiple studies, and Akt activation has been correlated with malignant proliferation and evasion of apoptosis in various cancers [22]. Accordingly, the Akt pathway is central to tumour development, and as a key player in apoptotic cascades, it offers a promising therapeutic target for the treatment of cancer [13, 23]. Jaceosidin treatments effectively inhibited Akt phosphorylation in the present OSCC cell lines, and ectopic activation of Akt diminished the proapoptotic effects of jaceosidin (Figure 6). Collectively, these results indicate that jaceosidin-induced apoptosis in OSCC cells is in part mediated by inactivation of the Akt pathway.

## 5. Conclusion

The present observations demonstrate that jaceosidin selectively inhibits OSCC cell proliferation by inducing caspase-dependent apoptosis and inhibiting the Akt pathway. We also clarified the modes of action of jaceosidin in OSCC cells and suggested that further consideration of jaceosidin as a promising new chemotherapeutic agent for the treatment of OSCC is warranted.

## References

[1] Jung K.-W., Won Y.-J., Kong H.-J., Oh C.-M., Lee D. H., Lee J. S. (2014). Cancer statistics in Korea: incidence, mortality, survival and prevalence in 2011.

[2] Jerjes W., Upile T., Petrie A. (2010). Clinicopathological parameters, recurrence, locoregional and distant metastasis in 115 T1-T2 oral squamous cell carcinoma patients.

[3] Mollaoğlu M., Erdoğan G. (2014). Effect on symptom control of structured information given to patients receiving chemotherapy.

[4] Wong R. S. Y. (2011). Apoptosis in cancer: from pathogenesis to treatment.

[5] Park E. Y., Lee K.-W., Lee H.-W. (2008). The ethanol extract from Artemisia princeps Pampanini induces p53-mediated G1 phase arrest in A172 human neuroblastoma cells.

[6] Chung K.-S., Choi J.-H., Back N.-I. (2010). Eupafolin, a flavonoid isolated from Artemisia princeps, induced apoptosis in human cervical adenocarcinoma HeLa cells.

[7] Kim M. J., Han J. M., Jin Y. Y. (2008). In vitro antioxidant and anti-inflammatory activities of jaceosidin from Artemisia princeps Pampanini cv. Sajabal.

[8] Na D. H. (1877). Metabolism study of botanical drugs.

[9] Song W. Y., Ji H. Y., Baek N.-I., Jeong T.-S., Lee H. S. (2010). In Vitro metabolism of Jaceosidin and characterization of cytochrome P450 and UDP-glucuronosyltransferase enzymes in human liver microsomes.

[10] Lee T. H., Jung H., Park K. H., Bang M. H., Baek N.-I., Kim J. (2014). Jaceosidin, a natural flavone, promotes angiogenesis via activation of VEGFR2/FAK/PI3K/AKT/NF-iB signaling pathways in endothelial cells.

[11] Lee H.-G., Yu K.-A., Oh W.-K. (2005). Inhibitory effect of jaceosidin isolated from *Artemisiaargyi* on the function of E6 and E7 oncoproteins of HPV 16.

[12] Chung K.-S., Choi H.-E., Shin J.-S. (2015). Chemopreventive effects of standardized ethanol extract from the aerial parts of *Artemisia princeps* Pampanini cv. Sajabal via NF-*κ*B inactivation on colitis-associated colon tumorigenesis in mice.

[13] Nitulescu G. M., Margina D., Juzenas P. (2016). Akt inhibitors in cancer treatment: The long journey from drug discovery to clinical use (Review).

[14] Liang F., Xie S. (2017). Puerarin prevents tumor necrosis factor-*α*-induced apoptosis of PC12 cells via activation of the Pi3K/Akt signaling pathway.

[15] Shu L., Cheung K., Khor T. O., Chen C., Kong A. (2010). Phytochemicals: cancer chemoprevention and suppression of tumor onset and metastasis.

[16] Min A. J., Ki W. L., Yoon D.-Y., Hyong J. L. (2007). Jaceosidin, a pharmacologically active flavone derived from Artemisia argyi, inhibits phorbol-ester-induced upregulation of COX-2 and MMP-9 by blocking phosphorylation of ERK-1 and -2 in cultured human mammary epithelial cells.

[17] Lee J.-G., Kim J.-H., Ahn J.-H., Lee K.-T., Baek N.-I., Choi J.-H. (2013). Jaceosidin, Isolated from dietary mugwort (*Artemisia princeps*), Induces G2/M cell cycle arrest by inactivating cdc25C-cdc2 via ATM-Chk1/2 activation.

[18] Lv W., Sheng X., Chen T., Xu Q., Xie X. (2008). Jaceosidin induces apoptosis in human ovary cancer cells through mitochondrial pathway.

[19] Khan M., Yu B., Rasul A. (2012). Jaceosidin induces apoptosis in U87 glioblastoma cells through G2/M phase arrest.

[20] Kiraz Y., Adan A., Kartal Yandim M., Baran Y. (2016). Major apoptotic mechanisms and genes involved in apoptosis.

[21] Rasul A., Yu B., Khan M. (2012). Magnolol, a natural compound, induces apoptosis of SGC-7901 human gastric adenocarcinoma cells via the mitochondrial and PI3K/Akt signaling pathways.

[22] Rasul A., Khan M., Yu B. (2013). Isoalantolactone, a sesquiterpene lactone, induces apoptosis in SGC-7901 cells via mitochondrial and phosphatidylinositol 3-kinase/Akt signaling pathways.

[23] Rasul A., Ding C., Li X. (2012). Dracorhodin perchlorate inhibits PI3K/Akt and NF-*κ*B activation, up-regulates the expression of p53, and enhances apoptosis.

